# ‘Breast Cancer Resistance Likelihood and Personalized Treatment Through Integrated Multiomics’

**DOI:** 10.3389/fmolb.2022.783494

**Published:** 2022-04-14

**Authors:** Sabba Mehmood, Muhammad Faheem, Hammad Ismail, Syeda Mehpara Farhat, Mahwish Ali, Sidra Younis, Muhammad Nadeem Asghar

**Affiliations:** ^1^ Department of Biological Sciences, National University of Medical Sciences, Rawalpindi, Pakistan; ^2^ Department of Biochemistry & Biotechnology University of Gujrat, Gujrat, Pakistan; ^3^ Department of Medical Biology, University of Québec at Trois-Rivieres, Trois-Rivieres, QC, Canada

**Keywords:** breast cancer, drug resistance, genomics, transcriptomics, proteomics, metabolomics, radiomics

## Abstract

In recent times, enormous progress has been made in improving the diagnosis and therapeutic strategies for breast carcinoma, yet it remains the most prevalent cancer and second highest contributor to cancer-related deaths in women. Breast cancer (BC) affects one in eight females globally. In 2018 alone, 1.4 million cases were identified worldwide in postmenopausal women and 645,000 cases in premenopausal females, and this burden is constantly increasing. This shows that still a lot of efforts are required to discover therapeutic remedies for this disease. One of the major clinical complications associated with the treatment of breast carcinoma is the development of therapeutic resistance. Multidrug resistance (MDR) and consequent relapse on therapy are prevalent issues related to breast carcinoma; it is due to our incomplete understanding of the molecular mechanisms of breast carcinoma disease. Therefore, elucidating the molecular mechanisms involved in drug resistance is critical. For management of breast carcinoma, the treatment decision not only depends on the assessment of prognosis factors but also on the evaluation of pathological and clinical factors. Integrated data assessments of these multiple factors of breast carcinoma through multiomics can provide significant insight and hope for making therapeutic decisions. This omics approach is particularly helpful since it identifies the biomarkers of disease progression and treatment progress by collective characterization and quantification of pools of biological molecules within and among the cancerous cells. The scrupulous understanding of cancer and its treatment at the molecular level led to the concept of a personalized approach, which is one of the most significant advancements in modern oncology. Likewise, there are certain genetic and non-genetic tests available for BC which can help in personalized therapy. Genetically inherited risks can be screened for personal predisposition to BC, and genetic changes or variations (mutations) can also be identified to decide on the best treatment. Ultimately, further understanding of BC at the molecular level (multiomics) will define more precise choices in personalized medicine. In this review, we have summarized therapeutic resistance associated with BC and the techniques used for its management.

## Introduction

Cancer is a common disease and represents one of the biggest health problems in the world and a significant global concern. The incidence and mortality rates of breast cancer (BC) have increased in recent years, and BC is currently the leading cause of cancer deaths in women worldwide.

According to the global cancer statistics in 2020, breast cancer (BC) in women was reported as the primary leading cause of deaths (Bray et al., 2018; WHO 2021). It occurs in every country of the world and in women of every age, although later years of life are an increased risk factor. According to the WHO fact sheet on breast cancer, in 2020, 2.3 million women were diagnosed with breast cancer, and 685,000 died from this cancer. As estimated at the end of 2020, almost 7.8 million women have been diagnosed with breast cancer in last 5 years (WHO 2021). This has made breast cancer the most prevalent cancer globally, and its prevalence has even surpassed lung cancer, which was previously the highest diagnosed cancer (Sung et al., 2021).

Breast cancer (BC) mainly has four molecular subtypes which have been defined in the large part of the hormone receptor or other forms of protein involved or not involved in each type of cancer: 1) luminal A or HR+/HER2– (HR-positive/HER2-negative) 2) luminal B or HR+/HER2+ (HR-positive/HER2-positive) 3) HER2-positive 4) triple-negative or HR–/HER2– (HR/HER2-negative) (Eliyatkın et al., 2015). This classification is mainly based on the type, behavior, and pattern of the cancer cells. A comprehensive understanding of all these types enabled the researchers and scientists to develop the targeted treatments and also the understanding that which type of treatment is best suited for which type of cancer cells (Sharma et al., 2010). Among all of the aforementioned types, the triple-negative subtype is the most prevalent and most aggressive as its response to chemotherapy is quite higher than that of the other types. Moreover, despite adjuvant chemotherapy, the survival rate of the patients with the triple-negative type is very poor (Anders and Carey 2009).

Chemoresistance is the major problem in the treatment and management of BC, when there is a relapse in the early-responsive tumors and development of resistance toward the multiple anticancer agents having various mechanisms and structures (Perez, 2009). Chemoresistance of tumors can be associated with multiple factors or mechanisms, which include its microenvironment, interaction with other cancer cells, modulation of immune cells and macrophages associated with cancer cells, cancer stem cells, and heterogeneity of cancer cells, that can modify the microenvironment of the cancer cells or tumors during chemotherapy which can lead to the development of resistance in them. There are several intrinsic factors contributing toward resistance development including the pH of cells, paracrine signaling among cells, and the hypoxia environment (Mansoori et al., 2017; Nikolaou et al., 2018). Another type of resistance toward multiple anticancer agents is known as multidrug resistance (MDR). However, the potential role of the drug-resistant genes that are involved in the transportation of anticancer agents is still unclear. Therefore, a clear understanding of the underlying mechanism of chemotherapy resistance and available treatments is required to develop successful strategies to overcome multiple drug resistance and other chemotherapy-associated resistances (Wind and Holen 2011).

There are many types of treatment therapies (surgery, chemotherapy, hormonal therapy, biological therapy, and radiation therapy) available depending upon the type of the cancer cell (Division of Cancer Prevention and Control, 2020). To choose the treatment for BC, there are certain modalities that need to be considered such as the location and size of the tumor, histopathology, lymph node commitment, presence or absence of metastases, and the molecular subtype of the cancer cells. Moreover, patient age, health, and hormonal status should be taken into consideration (NCI, 2022), Cardoso et al., 2019). Although chemotherapy has been used for the treatment of inflammatory and advanced-stage BC, there is a need to develop new strategies and predictive molecular markers to increase the prognosis of the patients (Cleator et al., 2007). The unpleasant side effects of the available breast cancer treatment methods motivate researchers to find some alternative options (Akram et al., 2017). The development of precision medicine is a great hope toward better breast cancer management. The precision medicine refers to the consideration of individual variations, environment, genes, and lifestyle for disease prevention and treatment (Collins and Varmus 2015). The recent advancement in the omics technology has allowed a more precise approach toward breast cancer treatment (Naito and Urasaki 2018). Moreover, the novel prognostic and predictive markers will be helpful in determining the patient that could benefit from the chemotherapy. In addition, different strategies can be defined to increase the targeted drug delivery response toward tumor cells which includes nanoparticles as well. These small nanostructures can be effective carriers not only in chemotherapy but also to overcome drug resistance as well (Lainetti et al., 2020).

## Breast Cancer Resistance Likelihood

Breast cancer is a very complex and heterogeneous disorder with unique molecular and morphological features relative to a disease which involves only a single gene or protein in a simple signaling pathway contributing toward the progression of disease in an independent and autonomous manner (Organization 2019). Various studies had represented BC heterogeneity through the differential response of the same type of BC patients to treatment and risk of developing side effects. One of the major clinical complications in the treatment of breast carcinoma patients is the development of therapeutic resistance (Luque-Bolivar et al., 2020). Recently drug resistance in BC treatment is not properly addressed, rather to focus on molecular pathways deeply; an alternative strategy of using a different drug is commonly applied. In order to reduce the adverse effects of BC treatment including drug resistance, a profound understanding of the molecular mechanism of the disease and the response to the drug is needed. Multidrug resistance (MDR) and consequent relapse on therapy are prevalent issues related to breast carcinoma as our understanding is incomplete related to the molecular mechanism of breast carcinoma disease (Waks and Winer, 2019a). Therefore, elucidating the molecular mechanisms involved in drug resistance is critical. For the management of breast cancers, the treatment decision not only depends on the assessment of prognosis factors but also on the evaluation of pathological and clinical factors. Integrated data assessments of these multiple factors of breast carcinoma through multiomics can provide significant insight and hope for making therapeutic decisions (Parsons and Francavilla 2020). Major BC treatment strategies rely on the tumor subtype, immunohistochemical evaluation of prognostic elements, and seek new genetic markers to improve the diagnostic strategies and to enhance treatment outcomes with minimal side effects.

## Conventional Breast Cancer Treatment Resistance

Endocrine therapy is included in one of the key conventional BC treatments along with chemotherapy and targeted therapy. For instance, it is used for treating tumors with positive hormone receptors (ER and PR) (luminal A and luminal B); however, chemotherapy is also required for some patients. Monoclonal antibody treatment is applied for HER2+ tumors (luminal B and HER2+). For positive hormone receptors, RNAi-mediated silencing and endocrine therapy are helpful (Tai et al., 2010; Harbeck et al., 2019; Waks and Winer, 2019b). The triple-negative breast cancer (TNBC) is only treated with chemotherapy. Various molecular players are being explored to study the BC cell resistance development to conventional therapies. Both intrinsic and extrinsic factors contribute toward creating resistance by BC cells, including its microenvironment, interaction with other cancer cells, modulation of immune cells and macrophages associated with cancer cells, cancer stem cells, and heterogeneity of cancer cells, that can modify the microenvironment of the cancer cells or tumors during chemotherapy which can lead to the development of resistance in them. There are several intrinsic factors contributing toward resistance development including the pH of cells, paracrine signaling among cells, and hypoxia environment. Another type of resistance toward multiple anticancer agents is known as multidrug resistance (MDR). A detailed overview of drug resistance to various BC subtypes and alternative approaches to overcome resistance is represented in Table 1. Here, out of many conventional treatment options, endocrine therapy is taken as a standard therapy for the treatment of ER+ BC, which includes the use of selective ER modulators, such as tamoxifen (TAM) (Group 2005; Cardoso et al., 2019) selective ER downregulators (fulvestrant, FUL), and aromatase inhibitors (AIs).

**TABLE 1:** Overview of drug resistance to various BC subtypes and alternative approaches to overcome resistance.

BC subtype	Treatment options (drugs)	Drug resistance	Resistance treatment options	References
*ER+*	A selective ER modulator, tamoxifen (TAM)	1. Mutations in estrogen receptor 1 (*ESR1*) gene and polymorphisms in cytochrome P450 family 2 subfamily D member 6 (*CYP2D6*) cause disruptions in TAM metabolism	Selective ER downregulator (fulvestrant, FUL) treatment is applied which has relatively low toxicity than TAM	(Kang et al., 2005
		2. Alterations in translation signals due to aberrant activation of cyclic adenosine monophosphate/protein kinase A (cAMP/PKA), mitogen-activated protein kinase (MAPK/ERK), and phosphatidylinositol 3-kinase (PI3K)/protein kinase B (AKT) signaling pathways		Li et al., 1997
		3. Mutations in the tumor suppressor protein, phosphatase, and tensin homolog (PTEN) may lead to activation of the PI3K/AKT pathway which causes TAM resistance		Group 2011
				Zakharchenko et al., 2011
				Razavi et al. (2018)
* ER+/PR+/HER2-*	selective ER downregulators (fulvestrant, FUL)	Mutations in the *PIK3CA* gene	Combination with Piqray (alpelisib)	(Thorpe et al., 2015
			(FUL + Piqray)	Administration (2019)
All clinical stages of BC	Aromatase inhibitors (AIs) (anastrozole, exemestane, and letrozole)	Relapse after initial treatment with a non-steroidal AI (anastrozole or letrozole)	Treatment with exemestane alone or in combination with an mTOR inhibitor such as everolimus	(Carlini et al., 2007
				Chin et al., 2007
				Geisler et al., 2008
				Bahrami et al. (2020)
* ER+/HER2-*	CDK4/6 inhibitors (palbociclib, ribociclib, and abemaciclib)	Uncomplicated and manageable hematological mainly neutropenia and non-hematological toxicities with dose interruption or reduction	Combination of CDK4/6 inhibitors with FUL and AI’s	(Asghar et al., 2015
				Turner et al., 2018
				Killock 2019
				Rossi et al. (2019)
* ER+*	*PI3K* inhibitors	Higher toxicity from the FUL + pictilisib combination treatment	Overcomes resistance to hormone therapy by controlling the AKT/mTOR signaling pathway by using everolimus (Afinitor) with the CDK4/6 inhibitor	(Hurvitz and Peddi 2013
	Pictilisib and buparlisib			Krop et al., 2016
				Dhakal, Antony Thomas et al. (2020)
* HER2+*	Humanized monoclonal antibodies (mAbs) trastuzumab, pertuzumab, and 19H6-Hu	Truncated form of HER2 (P95HER2) through proteolytic detachment created clinical resistance to trastuzumab	1.Pertuzumab is a second-generation recombinant humanized monoclonal antibody that binds to the extracellular dimerization domain II of HER2	(Warmerdam et al., 1991
		*PIK3CA* mutations	2. A new anti-HER2 antibody (19H6-Hu), which enhances the antitumor efficacy of trastuzumab and pertuzumab with a distinct mechanism of action	Christianson et al., 1998
		*FCGR*	3. PI3K/AKT/mTOR inhibitors along with trastuzumab or trastuzumab and paclitaxel are efficient and more safe	Quandt et al., 2011
		IIa polymorphisms		Administration 2019
				Zhang et al. (2020)
* TNBC*	Chemotherapy by alkylating agents, antimetabolites, anti-tumor antibiotics, topoisomerase inhibitors, TKIs, and mitotic inhibitors.	Alterations in the epigenetic mechanism	Epigenetic therapies, such as hydralazine and valproic	(Verweij et al., 1994
		Enzyme system that deactivates anticancer drugs	Neoadjuvant chemotherapy and taxanes along with anthracyclines	Miklavčič et al., 2014
		Tumor microenvironment, upregulation of *TWIST1* by NF-κB contributes to the chemoresistance	Immunotherapy	Loibl and Furlanetto 2015
			*ECT*	Jazieh et al. (2020)

## Overcoming Resistance Through Emerging Technologies

In recent years, advances have been made in BC treatment options including immunotherapy, cyclin-dependent kinase 4/6 (CDK4/6) inhibitors, tyrosine kinase inhibitors (TKIs), clustered regularly interspaced short palindromic repeats (CRISPR), microRNAs (miRNAs), use of already available drugs for different diseases for treating BC (drug repurposing), nanotechnology-based treatments, and electrochemotherapy (ECT). Additional benefits have been added to the conventional treatment options by introducing new strategies in terms of decreasing the side effects and overcoming resistance. Multiomics is the most recent emerging technology for treating BC through personalized decisions and treatment options. It usually generates a vast amount of data on different kinds including genomics, transcriptomics, proteomics, metabolomics, and radiomics. One of the biggest challenges is to integrate in data to obtain biologically meaningful insight (Wang et al., 2014; Nik-Zainal et al., 2016). For this purpose, researchers need robust and sophisticated computational systems to integrate and analyze the data in a standardized manner (Chen et al., 2017). This can be achieved by making improvements in technology for better results in less sample processing and measurement time.

The BC treatment decision not only depends on the assessment of prognosis factors but also on the evaluation of pathological and clinical factors. Integrated data assessments of these multiple factors of breast carcinoma through multiomics can provide significant insights and hope for making therapeutic decisions. The implementation of omics approaches including genomics, transcriptomics, proteomics, metabolomics, and radiomics in clinical practice will assist the analysis of global level patient’s changes which improves diagnosis and therapeutic choice on the basis of few markers (Scherf et al., 2000; Staunton et al., 2001; Bild et al., 2006). Early tumor detection will be facilitated by identification of omics technology-guided biomarkers, ultimately leading to early treatment and management of disease as marking the novel molecular targets confined to specific BC subtypes will decrease the reliance on non-targeted therapies, thus improving the quality of life for breast cancer patients.

## Multiomics Approaches and Breast Cancer Management

**FIGURE 1:**
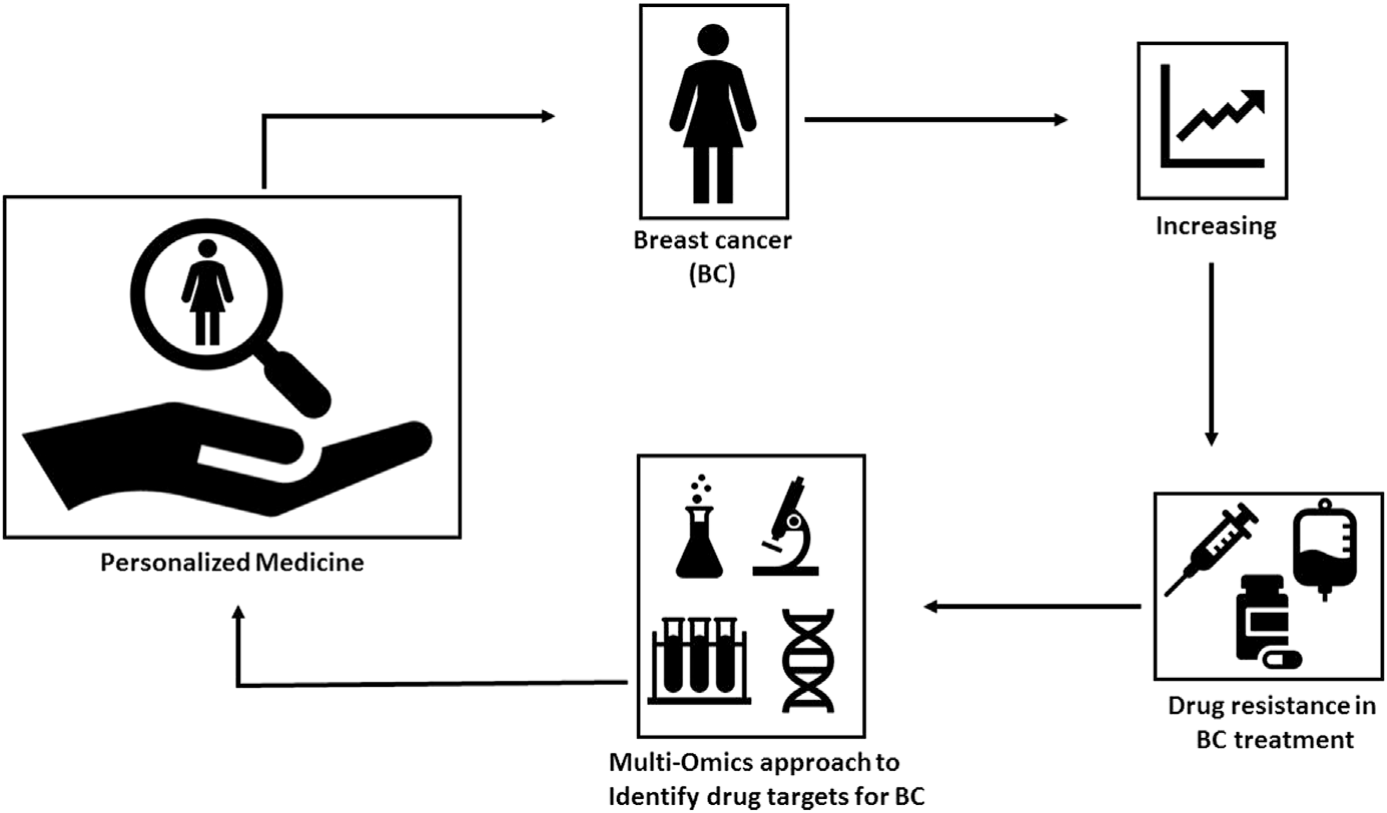
Multiomics approaches and breast cancer management.

Multiomics also described as panomics and/or integrative omics is an analytical approach that combines data from multiple ‘omics’ approaches including genomics, transcriptomics, proteomics, metabolomics, epigenomics, metagenomics ,and metatranscriptomics to answer the complex biological questions. This omics approach is particularly very helpful in identifying biomarkers of health, disease, and treatment progress by collective characterization and quantification of pools of biological molecules within and among the cells. A range of omics software and databases are available for this analysis. Omics techniques produce a large amount of the data which is then processed. Advanced technologies have allowed ‘omics’ data analysis in a combined, interconnected, and holistic format to solve the complex biological problems which could not have been found with experimental work in the laboratory Figure 1. Systems biology is an approach in biomedical research to understand the larger picture be it at the level of the organism, tissue, or cell by putting its pieces together. It is in stark contrast to decades of reductionist biology, which involves taking the pieces apart.

## Genomics and Breast Cancer Management

NGS has allowed rapid DNA sequencing covering the whole genome. This approach helped in redefining the breast cancer subtypes and identification of mutations and SNPs as biomarkers for BC management (Parsons and Francavilla 2020). Additionally, single-cell investigation allowed the study of BC stem cells as a novel therapeutic approach (Lawson et al., 2015). Genomics has started to change the trend of BC treatment. Genomics with molecular signatures deescalated chemotherapy and personalized treatments of BC. Molecular signatures play vital roles in the prediction of therapeutic targets. In BC, key signatures are the PR (progesterone receptor), ER (estrogen receptor), and HER2 (human epidermal growth factor receptor 2) (Rakha et al., 2010). For management, if a patient is PR+ or ER+ will probably receive endocrine treatment, while HER2 patients will likely receive trastuzumab. Triple-negative breast cancer (TNBC) covers all types of tumors which are PR-, ER-, and HER2-negative. TNBC is a more aggressive tumor and is associated with a poorer outcome to chemotherapy. However, there is still no targeted therapy for TNBC (Foulkes et al., 2010).

In the context of hereditary predisposition, the United States National Comprehensive Cancer Network proposed 19 genes as clinical screening tests for BC, while the Genetics and Cancer Group proposed 13 genes for prevention and screening measures (Hamdan et al., 2019). Genome sequencing enables the discrimination of genetic modifications on the basis of *TP53*, *PIK3CA*, and *GATA3* genes, and results suggested that these genes are modified in more than 10% of BC patients. On the other hand, NGS revealed that BC generally carries mutations in the *TP53*, *BRCA1*, and *RB1* genes (Koboldt et al., 2012). It is estimated that *BRCA1* mutations chances are in 10% of patients. However, in young females, TNBC chances are 20% (Peto et al., 1999). *BRCA1* mutations do not account for all inherited BC cases associating the existence of other genes (Ellsworth et al., 2010). *BRCA1* and *BRCA2* identification opened the paths for screening tests to identify different mutation points for hereditary BC. For early age diagnosis, BC screening is now recommended for females with a family history of cancer (Nelson et al., 2005). Currently, BRACAnalysis^®^ is the sole sequencing provider for the detection of mutations in *BRCA1* and *BRCA2* (Ellsworth et al., 2010).

A meta-analysis of genomic studies recognized 84 loci, probably associated with the risk of BC including lymphocyte-specific protein (LSP1), fibroblast growth factor receptor-2 (FGFR2), mitogen-activated kinase-1 (MAP3K1), and trinucleotide repeat containing 9 (TNRC9/LOC643714) (Ellsworth et al., 2010; Michailidou et al., 2015). Along with it, several low penetrance variants were also identified without any validation. One such variant is the FGFR2 oncogene whose protein is being highly expressed in 5% of BC patients. This refers to SNP which affects the target binding site of FGFR2 and activates the additional downstream pathway (Moffa and Ethier 2007). Similarly, another SNP, in the 8q24 region, regulates the C-MYC oncogene (Ahmadiyeh et al., 2010).

Ki67 is another proliferative biomarker that is currently being used to predict the growth rate of tumor (Lal et al., 2017). The combination of these four signatures (ER, Ki67, PR, and HER2) is referred to as a protein-based ‘signature’. On the basis of this panel, different algorithms have been developed for the prediction of the BC recurrence risk. Several models have been validated to enhance the BC management with a combination of pathological, clinical, and biosignature data. Numerous tools have been designed (e.g., Predict, Online, Adjuvant!, and the Nottingham Prognostic Index) to help clinicians with patients’ treatment decision about adjuvant therapy or surgery. These tools incorporate various pathological and clinical variables together with the tumor expression of these molecular signatures (ER, Ki67, PR, and HER2) to predict survival with or without adjuvant therapy (Bartlett et al., 2016). In spite of recent achievement in identifying genetic biomarkers with additional low-risk alleles and low frequency, highly-incident variants (Bodmer and Bonilla 2008) and environmental interactions with genes must be evaluated, and methods must be established to assess mechanisms by which DNA variants in intronic or intergenic regions contribute to BC (Ellsworth et al., 2010).

## Transcriptomics and Breast Cancer Management

The study of the complete set of RNA molecules that are produced by the genome under specific conditions in specific cell/tissue using modern techniques, e.g., microarrays and RNA-Seq, fall under the umbrella of transcriptomics. Transcriptomics has been widely used to investigate biomarkers for BC’s risk assessment, subtype identification, disease progression, survival, and invasion that could be subsequently utilized to assess treatment success and clinical trials (Transcriptome 1–4). In the breast cancer treatment, biomarkers are crucial as prognostic or predictive properties. Based on the values of these biomarkers, BC treatment which could be hormonal therapy, chemotherapy, and molecular targeted therapy is planned. Transcriptomics has assisted a lot in the discovery of the BC’s biomarkers. In the subsequent section, we have discussed a few of the biomarkers that have been discovered and are used for BC’s management.

Transcriptome-wide association studies (TWAS) combine the data from whole genome sequencing and microarray or RNA-Seq to get insights into the BC’s management. Mancuso et al. identified 1,196 genes that were associated with 30 complex biological pathways in BC using the TWAS approach (Mancuso et al., 2017). At present, three TWAS studies have been reported by different groups. Gao et al. reported TP53INP2 (tumor protein p53-inducible nuclear protein 2) to be efficiently linked with ER-negative BC in all three studied populations, i.e., African, European, and Asian ancestry populations (Gao et al., 2017). Similarly, Hoffmann et al*.* identified significant links between the BC risk and the expression of RCCD1 (RCC1 domain containing 1) and DHODH (dihydroorotate dehydrogenase) in the breast tissue, along with association with ANKLE1 (Ankyrin Repeat and LEM Domain Containing 1) in trans-ethnic meta-analyses of U4C, and UK Biobank data were elucidated (Hoffman et al., 2017). Wu et al. identified 48 genes from which 14 were novel using the data acquired for the Genotype-Tissue Expression Project. The effect of these genes on cell proliferation and colony-forming efficiency was elucidated to provide insights into the BC biology (Wu et al., 2018). Another group identified 26 new target genes for breast cancer including 17 genes for estrogen receptor (ER)-negative BC using expression quantitative trait loci (eQTL). Furthermore, seven regions with variants linked with BC risk and four regions for ER-negative BC risk were also identified *via* gene-based test of linkage that considers eQTL from multiple tissues. However, the function of most of these genes was not known (Ferreira et al., 2019). These studies have reported 59 genes whose predicted expression levels are associated with a high risk of BC. Additional five genes are associated with the ER-disease risk. Of these 64 genes, 30 are at loci that were not previously identified by breast cancer GWAS.

Feng and co-workers identified two genes, *HIST2H2BA* and *STXBP4*, which were precisely associated with ER+ but not with ER- BC through meta-analysis using publicly available data for whole transcriptome and genome sequencing from the GTEx database. Furthermore, 26 old and four novel biomarkers were also identified that were associated with BC’s risk (Feng et al., 2019).

Currently, six tests including the Breast Cancer Index, EndoPredict, MammaPrint, Oncotype DX, Prosigna, and genomic grade index have been designed on the basis of the transcriptomic signatures for early diagnosis of the BC. The breast cancer index is designed on 60 ER+ tumor samples from patients previously treated with tamoxifen. It measures the ratio of *HOXB13* and *IL17BR* genes together with expression of the genomic grade index genes including *BUB1B*, *NEK2*, *CENPA*, *RRM2*, and *RACGAP1*. This test is used to determine the prognosis of the women with estrogen receptor-positive and lymph node-negative disease (Ma et al., 2008; Jerevall et al., 2011). The EndoPredict is designed on 964 ER+ tumor samples from patients with LN ± disease treated with tamoxifen. This test includes the expression of eight tumor-associated genes including *BIRC5*, *UBE2C*, *RBBP8*, *AZGP1*, *IL6ST*, *MGP*, *DHCR7*, and *STC2* and three control genes *OAZ1*, *CALM2*, and *RPL37A*. This test is used in determining the prognosis of women with estrogen receptor-positive and lymph node ± disease (Filipits et al., 2011).

MammaPrint is a 70-gene test which uses microarray technology for quantitative expression of the genes belonging to the following processes: cell-cycle dysregulation (15 genes), angiogenesis (12 genes), proliferation and oncogenic transformation (11 genes), invasion and metastasis (8 genes), growth factor signal transduction (6 genes), resistance to apoptosis (2 genes), and miscellaneous/unknown function (16 genes). This test has been designed on 78 ER ± tumor samples with a diameter. This test determines the prognosis of women with ER ± and LN− disease of stages 1 or 2. This assay was approved in 2007 by the FDA to predict the risk level of a patient for developing metastasis. (Verweij et al., 1994; Van’t Veer et al., 2002). Oncotype DX has been evaluated on 447 ER ± tumor samples from patients with LN ± disease registered in three distinct clinical trials, including from the tamoxifen only the arm of NSABP B-20. This test measures genes for proliferation (5), invasion (2), estrogen (4), HER2 (2), GSTM1, BAG1, CD68, and also five genes for reference. It is used to predict 10-year recurrence risk in patients with ER+ and LN− disease (Paik et al., 2004). The Prosigna test is designed on 189 ER ± tumor samples from patients with LN ± disease and 29 nonmalignant breast tissue biopsy samples. This test measures the expression level of 50 genes along with five reference genes to classify BC into one of four intrinsic subtypes. Clinically, it has been utilized to also determine the prognosis of postmenopausal women with ER+ and LN ± disease of stages 1 or 2 (Parker et al., 2009; Nielsen et al., 2010) Although ample work has been done on the discovery of the biomarkers for BC’s diagnosis, progression, and treatment end point, further investigations are required to identify the biomarkers for diverse forms of the breast cancer.

## Proteomics and Breast Cancer Management

Proteomics is the fine study of complete set of proteins present in any tissue, cell, or organism. Breast cancer (BC) proteomics research is based on validating and discovering protein predictive biomarkers diagnostic purposes. Recently, a study of four groups reported the survival patterns of BC functional proteins (Korkola and Gray 2010) which revealed about 10 different protein biomarkers that might differentiate BC subgroups biologically and clinically more accurately as compared to prognostic markers. Umar et al. (2005) identified nine tryptic peptides being differentially expressed by stromal and tumor analysis using laser capture microdissection. Afterward, Sanders et al. (2008) reported the reduced expression level of S100-A8 and ubiquitin in BC tissue than that in normal tissue.

Mass spectrometry analysis of the BC proteome revealed that protein-specific patterns are responsible for early diagnosis. 14-16 MS analysis also identified different peptide biomarkers including fragments of C3, C3adesArg, factor XIIIa, ITIH4, FPA, apoA-IV, fibrinogen, bradykinin, and transthyretin. These biosignatures can be used as a landscape for the early diagnosis of BC. Palacios et al. (2008) reported 37 protein biomarkers using proteomics classification. Among these, BRCA2-mediated cancers are found to be associated with the D1 and D3 cyclins along with CDK4. In another study, using protein markers and signaling pathways, five subtypes of ER-positive BC have been reported consisting of normal, basal, overexpressed HER-2, luminal A, and luminal B (Reis-Filho and Tutt 2008; Qin and Ling 2012; Zeidan et al., 2015). Collectively, 97 BC biosignatures have been reported so far from pathological and proteomics studies including ER, p53, CK8/18, Ki-67, PR, cyclin D1, HER-2, CK5/6, cyclin E, BCL2, cyclin E, and E-cadherin (Bhargava et al., 2008; Qin and Ling 2012; Zeidan et al., 2015). In another proteomic study, scientists have reported the role of retinoic acid receptor alpha as a potential biosignature in 28 ER-positive patients. Brozkova et al. identified the proteomic role of HSP27 and ANXV as biomarkers in BC 21, 22. He et al. (He et al., 2013) by using MS and ELISA reported that serum CD14 could be an active biomarker for the prediction of BC.

Kabbage *et al.* reported the overexpression of the Hsp27 and Hsp5 in BC tissues which are known as α-B-crystallin. Moyano *et al* (Moyano et al., 2006) reported that α-B-crystallin can solely be responsible for cancer transformation because it can induce the expression of EGF and anchorage-independent growth. α-B-crystallin has the ability to enhance cell invasion and migration along with the activation of the MAPK/ERK pathway. These reports suggest the oncoprotein nature of α-B-crystallin. Li et al. (2005) investigated three protein signatures including one reducing protein (4.3 kDa) and two increasing proteins (8.1 and 8.9 kDa) for BC. Studies revealed that structurally these proteins consist of the ITIH4 chain, C3adesArgΔ8 peptide, and C3adesArg (Belluco et al., 2007).

Hudelist et al. (2006) performed MALDI-TOF and 2-DE comparative analysis of LCM from normal and tumor tissues of five BC patients. In normal tissues, proteins with high MW and low isoelectric points were expressed in the extracellular matrix, while in LCM tissues, proteins with intermediate MW and high isoelectric points were overexpressed. Collectively, 32 proteins were expressed differentially and identified as tumor-suppressor genes, cytokines, signal-transducers, structural proteins, and cell-cycle regulators. Some proteins suggest their active role in tumor suppression as they are subregulated during cancer invasion including Maspin, DCC, and DSG3. On the other hand, CATH, HER-3, and HSP-27 are overexpressed during cancer invasion. Some overexpressed proteins such as CGG3 have a significant role in malignant transformation in BC also termed as ALADIN (Fink-Retter et al., 2009).

Pietrowska et al. (2010) reported proteome analysis in frozen LCM of breast tumor using MALDI MS. He compared protein expression in ER-negative and ER-positive tumors along with invasive carcinoma in mammary epithelium. Biosignatures were identified using appropriate statistical models and classifiers were validated in blinded tests. They used LC-MS/MS for identification and IHC for the confirmation of m/z features of the classifiers. A group of scientists compared the level of ubiquitin and calgranulin-A in 167 normal tissues with 122 tumor tissues, and it was found that ubiquitin expression was decreased while the expression of calgranulin-A was enhanced in tumor tissues. This study led to the identification of three biosignatures for BC. Schulz et al. (2009) reported the proteomic expression of TNBC compared with Her-2 positive tumors using MALDI-TOF/MS and 2D-DIGE. Through this technique, vimetin, L-plastin, glycolytic enzymes, fironectin, cytokeratins, annexin-1, annexin-2, and peroxiredoxin proteins were identified and validated by IHC and Western blotting.

Due to progresses in several genetic approaches, the development of BC diagnosis and treatment has been accelerated. Although the development and validation of molecular assays remained deficient for BC detection and preclinical decisions (Zakharchenko et al., 2011), progress in this regard is fundamentally required for the rapid management of BC.

## Metabolomics and Breast Cancer Management

One of the recent promising research areas in treating BC is metabolomics, which focuses on the study of metabolites and their metabolic pathways, which are quite different from the normal cell pathways. Metabolism can be studied in two ways: targeted and untargeted. Metabolomics databases are used to interpret metabolomics data through various bioinformatics tools such as mass spectrometer (MS) combined with chromatography and nuclear magnetic resonance (NMR) through which metabolic fingerprints and profile of specific samples can be generated (Cheung et al., 2019).

Metabolomics data generated can be applied to hunt for novel molecular biomarkers involved in BC prognosis, to monitor their metastatic state, drug response, and in making therapeutic decisions for BC management. Metabolomics is emerging fast in precision medicine, by which a personalized treatment is designed for a specific patient according to the patient’s molecular abnormalities represented by the metabolomics profile and fingerprints. Likewise, through pharmacometabolomics, drug response can be studied in patients by keeping their metabolic profile in view (Xu et al., 2012; Peng et al., 2015; Marshall and Powers 2017).

Recently, diet-related metabolites are extensively explored to relate with the risk of BC development and susceptibility (Playdon et al., 2017). This study represents various diet circulating metabolites can be robust and informative such as tocopherols (vitamin E), butter-related caprate, alcohol-related metabolites medium-chain SFA, fried food–related 2-hydroxyoctanoate, an odd-carbon MUFA, a hydroxy fatty acid, animal fat metabolites, and dessert-related g-CEHC can be associated with the risk of BC development in ER+ cases (Key et al., 2006; Playdon et al., 2017). After exploring metabolomics profiles and related pathways, it is suggested that the mechanism of developing BC through diet-related metabolites include alteration in various physiological processes such as the tumor suppression, immune function, and response to growth factors by breast cells, estrogen synthesis elevation in adipose tissues, and inflammation (Sczaniecka et al., 2012; Johnson et al., 2013). It is also observed by candidates’ dietary biomarkers which drive that androgen-dependent and androgen-independent mechanisms may induce alcohol-related BC particularly in postmenopausal ER+ cases.

Overall, studying diet-related metabolites and exploring the metabolic profiles of BC patients can give significant insights for developing dietary guidance for breast cancer prevention. However, challenges still exist in upgrading the technology for integrating such big data including metabolomics with other omics datasets. Nonetheless, metabolomics can play a crucial role in breast cancer diagnosis, understanding the molecular mechanism, and time management.

## Radiomics and Breast Cancer Management

Radiomics is an emerging field, and it provides quantitative and qualitative imaging biomarkers for the diagnosis, staging, distant-metastasis detection, therapeutic and prognostic prediction, and evaluation of therapeutic responses of BC. It is a method that uses data from clinical radiographic images through data-characterization algorithms and interprets the information by advanced computational analyses. PET/CT and MRI scans are widely used to evaluate the BC’s diagnosis, progression, and treatment success. Recent studies have proven that the combination of these techniques is more effective than individual ones. The clinical application use of PET/MRI/CT scans in detection of primary breast cancer is effective. Although PET, CT, and MRI scans have been used in the diagnosis of the primary breast cancer, however, their sensitivity and specificity differs depending upon the type of the breast cancer. The use of these technologies in different studies and their outcomes has been summarized by Ming et al. (2020). Conclusively, localized breast cancer can be better diagnosed with PET and MRI, while axillary and extra-axillary nodal metastases have been better diagnosed by combining PET/CT or PET/MRI. Additionally, PET/CT is superior in terms of monitoring local recurrence.

Radiogenomics is an emerging field of radiomics, which combines the information from clinical images and genomic databases using artificial intelligence for BC type determination, treatment plan, and outcome measurements. The integrated process of radiogenomics, crucial strategies, and statistical algorithms involved in current studies has been summarized by Shui et al. (2020). The application of radiogenomics in breast cancer, challenges, and future perspectives have been discussed in detail by Pinker et al. (2018). Although international guidelines, workflow, and standard procedures need standardization, this new field provides hope for atomization of the whole process.

### Nanobiotechnology and Breast Cancer Management

Nanotechnology offers numerous approaches for imaging, monitoring, diagnosing, and delivering chemotherapeutic drugs to the tumor site. Nanoparticles aid in providing medications with improved efficacy, lower toxicity, and the ability to bypass biological barriers, resulting in improved anticancer activity (Jain et al., 2020). Medication-loaded nanoparticles, micelles, and liposomes are examples of nanomedicine that have unique properties that allow them to pass through biological membranes and deliver the encapsulated drug to the cells. Nanotechnology has several distinguishing features, including small size (nanometric), active and passive targeting, the capacity to connect several targeting moieties, controlled release, and site-specific targeting. Particle size, shape, and surface chemistry are all characteristics that influence the cellular uptake, biodistribution, and clearance mechanisms (He et al., 2010). For diverse types of BC treatment, many other nanoparticulate chemotherapeutic delivery platforms have been in clinical trials (Alyassin et al., 2020).

Recent advances in nanoparticles imply that they could be used to target drugs selectively in BC without damaging normal cells and tissues. Reduced toxicity, biocompatibility, ease of manufacturing, and high encapsulation efficiency are all properties of nanoparticles. Nanoparticles have the advantage of isolating the medicine or encapsulated molecules from exposure to an external environment, which protects the drug while also protecting nearby cells (McClements 2018). To target Engrailed-1 (EN1), which is overexpressed in TNBC, Sorolla et al. produced a docetaxel-PGMA-PAA-nanoparticle conjugated with the peptide (EN1-RGD-iPep) (Jain et al., 2020). The results showed that employing peptide-functionalized nanoparticles inhibited cell proliferation and increased apoptosis. In addition, the dose of DTX encapsulated in nanoparticles was lowered from 20 mg/kg to 2 mg/kg. In T11 and SUM149 mouse models, peptide-conjugated nanoparticles improved antitumor activity, reduced tumor volume, and improved bioavailability and pharmacokinetics (Selot et al., 2016; Sorolla et al., 2019). In a separate investigation, PTX-loaded PEG-maleimide-fractionalized PLGA nanoparticles were coupled with an antibody for enhanced therapeutic efficacy in TNBC against perlecan (maintains endothelial barrier function). This work demonstrated that nanoparticles could improve tumor drug delivery to TNBC by showing increased cellular uptake, enhanced cytotoxicity, and tumor size reduction in these carriers. Classic medications such as gemcitabine and bevacizumab are used to treat a variety of malignancies, including ovarian, prostate, and breast cancers. In phase II therapeutic trial, gemcitabine and PTX-loaded albumin-stabilized nanoparticles were functionalized with bevacizumab mAb to decrease tumor development in BC patients. The study’s outcome was 6-month progression-free survival (PFS). In another clinical trial, albumin nanoparticles bound with rapamycin were used to treat recurrent breast cancer and showed therapeutic efficacy with a 5-year patient survival rate. Inhibition of the mTOR pathway was the primary mechanism for tumor growth regression (Khanna et al., 2019).

### Role of Nanobiotechnology to Overcome Multidrug Resistance in Breast Cancer

Anticancer medications encapsulated in nanoparticles can target tumor cells actively or passively, improving the therapeutic efficacy at the target region. Chemotherapy medications’ systemic toxicity can be reduced, and certain types of multidrug resistance can be avoided (Yuan et al., 2016). Passive targeting occurs when nanoparticles travel through holes in leaky blood arteries and are held by the aberrant draining lymphatic system, resulting in the EPR effect (increased permeability and retention). Passive targeting can occur when positively charged nanoparticles electrostatically interact with the negatively charged sialic acid and phospholipids on the surface of tumor-associated endothelial cells (Palakurthi et al., 2012). Active biomolecules such as nucleic acids, peptides, sugars, and antibodies can modify nanoparticles to bind to cancer cells actively (Yuan et al., 2016; Sorolla et al., 2019). Ideally, high-affinity nanoparticles combine specifically with molecules such as carbohydrates, proteins, folate, transferrin, aptamers, or lipids. Nanoparticle delivery minimizes damage to non-cancer cells because these are overexpressed on the surface of cancer cells. Active targeting is used to recognize cells precisely using functional biomolecule interactions, improve drug endocytosis by the cell, reduce cytotoxicity in non-cancer cells, raise drug concentration, and leverage the EPR effect (Ali et al., 2021). Once the cell is discharged into the cytoplasm, nanoparticles are taken in by the cell via endocytosis, frequently bypassing and avoiding old ABC-transporters responsible for lethal drug efflux (Choudhury et al., 2019). Clathrin-mediated endocytosis, caveolae-mediated endocytosis, macropinocytosis, and other endocytosis are the four primary mechanisms of endocytosis (Yuan et al., 2016). Apart from receptor-independent endocytosis, clathrin-mediated endocytosis is the best researched mechanism involved in receptor-mediated nanoparticle uptake. The transferrin, low-density lipoprotein (LDL) receptor, and epidermal growth factor receptors (EGFR), particularly the human epidermal growth factor receptor 2 (HER2), are all significant receptors (Patra and Turner 2014; Cerqueira et al., 2015). Nanoparticles that enter cells by clathrin-mediated endocytosis end up in lysosomes, where the acidic environment degrades them to release medicines (Malinovskaya et al., 2017). After nanoparticles bind to the cell membrane, caveolae-mediated endocytosis produces cytosolic caveolar vesicles. Folic acid, albumin, and cholesterol are ligands linked to caveolae-mediated endocytosis. Another nonselective endocytosis mechanism, macropinocytosis, is based on action-driven membrane protrusions that fuse with and separate from the plasma membrane to produce macropinosomes. In addition, nanoparticles encapsulating P-gp inhibitors and anti-cancer medicines can be employed to circumvent P-gp mediation MDR. Wong and colleagues mixed doxorubicin and elacridar, a P-gp inhibitor, in polymer-lipid hybrid nanoparticles. According to the findings, the simultaneous delivery of the two medications improved the treatment of multidrug-resistant breast cancer *in vitro* (Cerqueira et al., 2015; Yuan et al., 2016; McClements 2018; Sorolla et al., 2019).

### Precision Medicine

The recent developments in the “OMICS” sciences have uncovered many cellular mechanisms. This has led to the foundation of a new field of study known as precision or personalized medicine. It has been an established fact now that not all the cancer patients respond to the same treatment regime equally. The difference in treatment responses is subjected to the variation in the genome that each individual carries. These variations lead to the variations in responses toward the drugs. Precision medicine is changing the healthcare pattern by linking individual genetic information to drug applications, thus changing the conventional practice of medicine.

### Traditional Standards

**FIGURE 2:**
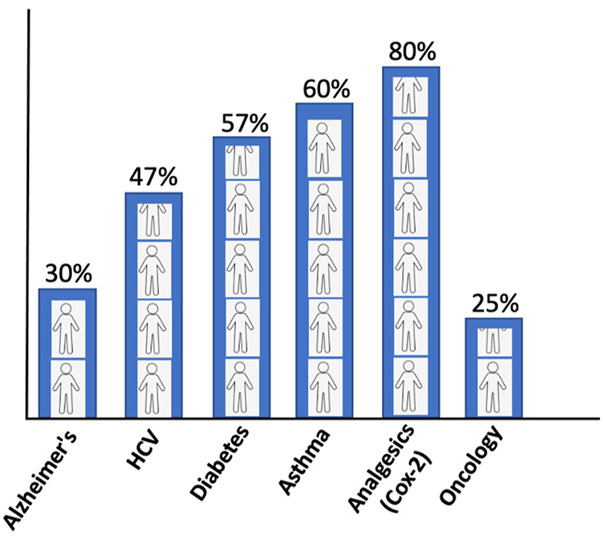
Patients’ response rate to major drugs against different diseases (Spear et al., 2001).

The conventional standard where the treatment strategy was “one-dose-fits-all” has been ineffectual as it incurs all the risks of the following drug toxicities and treatment failures. The drug inefficacy has been observed in several patients for different diseases. The variation in failures is 38–75%, where patients have no effect of the drug (Figure 2). In cancer drugs, the response rate is as low as 25% (Spear et al., 2001).

The adverse reactions of drugs as a treatment consequence are another problem. In the United States, 16% have shown adverse reactions to drugs. Meta-analysis has revealed that around 6.7% of all the patients admitted in the hospitals of the United States are associated with adverse drug reactions with more than 100,000 death reports annually (Lazarou et al., 1998; Spear et al., 2001). This makes precision medicine an advanced approach to the future medicine.

### Precision Medicine and Breast Cancer

Breast cancer is the primary cause of cancer in women globally (Sung et al., 2021). Recently, molecular investigations have shown that it is a combination of several diseases with various biological behaviors rather a single disease. Thus, precision medicine is the best choice in such circumstances. This new approach of oncology is utilized at different levels of breast cancer management, which includes treatment efficacy prediction, prognosis, and development of new treatments through new types of clinical trials. These trials would not only include the breast cancer targeting but also characterization of tumor genetics *via* advanced molecular genomics techniques such as next-generation sequencing. The aim of the precision medicine is to customize treatment according to the disease specificity for a given patient.

### Preventive Strategy

Precision medicine digs into the personal genetic and protein profiles to improve the healthcare at a more personalized level, with the help of the recently emerging ‘‘-omic’’ technologies that include genomics, proteomics, metabolomics, and pharmacogenomics (Tebani et al., 2016; Ahmed 2020). These techniques have enabled us to forecast the disease or its presence before the clinical symptom’s appearance. It enables us to act on the disease through an early intervention that can save lives in many cases. The analysis of the molecular characteristics of primary stage breast cancer using next-generation sequencing has led to the portrayal of the genomic background of the disease (Stephens et al., 2012). Such information is key for designing preventive schemes. For example, females carrying genetic mutations in the genes *BRCA1* or *BRCA2* have a greater chance of developing breast cancer (Riis 2021). Similarly, mutations of *TP53* and *PIK3CA* are the frequent genomic alteration in all intrinsic subtypes (Stephens et al., 2012). Other mutations are less frequent, but could be clinically relevant, that includes mutations and deletions in *PTEN*, *RB1,* and *AKT1* mutations. Sequencing information has identified mutations in other genes of interest that might be clinically relevant in breast cancer such as *KRAS*, *NF2*, *SKT11*, *APC*, and *AKT2*. A precise test of these breast cancer-associated genes can guide examination and preventive treatment based on the objective risk measurement such as the increased frequency of prophylactic surgery, chemoprevention, and mammography (Cui et al., 2014).

### Prediction Strategy

**FIGURE 3:**
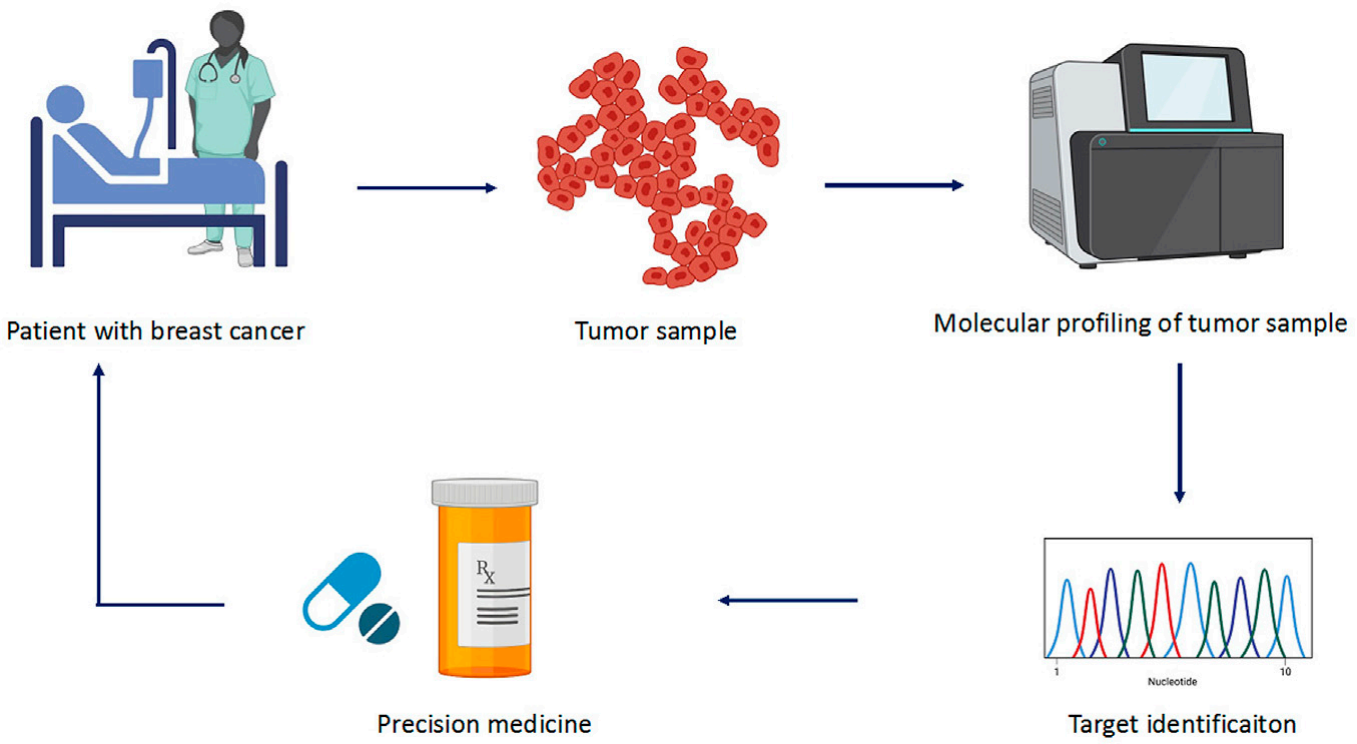
Different steps involved in precision medicine. The sample is collected from patients who are subjected to molecular profiling or high-throughput sequencing. The collected data are analyzed for mutation and target genes involved in the cancer. Based on the identified target, precise drug is selected to treat the cancer.

Precision medicine facilitates physicians to opt for therapies which are best suitable for the patients (Figure 3). This allows avoiding the adverse drug reactions. The molecular diagnostic devices used for the detection of predictive biomarkers provide vital information regarding the genetics of the patients who will benefit from a defined therapy. This type of subtyping is also applied in early breast cancer for the determination of the modality and decision for antitumor agents that best suit the patient (Sabatier et al., 2014). There are several multigene assays present that estimate the associated risks (Table 2). For example, MammaPrint (Agendia, Netherlands) utilize samples to examine 1,391 genes *via* microarray assays, and the results of 70 gene expression profile are used to assess the risk and classify patients in high to low risk for relapse (Van’t Veer et al., 2002; Van De Vijver et al., 2002). Oncotypes DX (Genomic Health, United States) is another generally used multigene assay. It uses a 21-gene signature (includes five reference genes) to conclude whether the patient with a certain breast cancer type would benefit from chemotherapy (Hornberger et al., 2005; McVeigh et al., 2014; Dafni et al., 2019). PMA50-based Prosigna (NanoString Technologies) analyzes a signature of 50 genes to assess the risk of recurrence (ROR) (Lænkholm et al., 2018). Similarly, EndoPredict (Myriad Genetics) is another breast cancer prognostic test. It does RNA expression analysis of eight target genes along with three normalization genes and a control gene. This information is used to predict ROR in patients with breast cancer at 10 years (Sestak et al., 2019). These diagnostic tests lead to the patient’s classification into subgroups that help physicians to make a treatment decision whether hormone therapy alone would be sufficient or may require chemotherapy. These assays are currently employed in practice guidelines and been utilized in the clinic.

**TABLE 2:** Diagnostic devices used for breast cancer multigene assays.

Assay	Sample	Number of genes	Analysis	Company
MammaPrint	Fresh/frozen paraffin-embedded (Fresh/frozen)	70	Microarray	Agendia (Netherlands)
Oncotype DX	Fresh/frozen paraffin-embedded	21	qRT-PCR	Genomic Health (US)
PAM50	Fresh/frozen paraffin-embedded (Fresh/frozen)	55	Microarray/qRT-PCR	ARUP Laboratories (US)
EndoPredict	Fresh/frozen paraffin-embedded	11	qRT-PCR	Myriad (US)

### Targeted Strategy

In recent times, molecular medicine has advanced, which is the key to precision medicine. In breast cancer, many potential druggable mutations have been determined. However, there is comparatively less proof to support the usage of matched molecular targeted agents in breast cancer. Mutation in *PIK3CA* occurs in approximately 25% of breast cancer, and it is reported as a driver of this disease (Cancer Genome Atlas Network 2012). However, in the early clinical trials, the use of the non-selective *PI3K* inhibitors led to modest response rates (4%) while administered as a monotherapy (Mayer et al., 2014), whereas the second-generation (α-selective) *PI3K* inhibitor produces improved inhibition *in vivo* in animal models and is more specific (Fritsch et al., 2014). Although initial results with these new inhibitors showed partial responses of about 6% in patients with *PIK3CA* (mutant) breast cancer, no responses had been detected in patients with *PIK3CAI* (wild-type) tumors (Janku et al., 2015). Therefore, it is important to design drugs which can hit the target with maximum specificity and bioactivity. The presence of mutation (two or more) in cancer-associated genes has been linked with resistance to targeted therapies *in vitro* and in clinical trials (Arnedos et al., 2015). It has been reported in the breast cancer that 67% of samples analyzed carry two or more genomic mutations. There are many such observed issues in breast cancer, for example, the co-existence of mutations of *PIK3CA* and amplification of *ERBB2* have been linked with resistance to *HER2*-targeting drugs lapatinib and trastuzumab (Fontanella et al., 2014), which provides the rationale of combining *HER2* and *PI3K* inhibitors in different clinical trials. However, such resistance toward anti-*HER2* therapy with the presence of *PIK3CA* mutations was not being observed when double blockage for *HER2* was attained via the usage of two monoclonal antibodies (Baselga et al., 2014). Cancer itself is also subjected to evolve through genetic diversification in a complex pattern. Therapeutic interventions might reduce or control cancer, but it might also stimulate the development of resistant variants (Wang et al., 2014; Eirew et al., 2015), such as tumor adaption via initiation of alternative protein networks which will dodge targeted inhibition. Such a mechanism has been reported in patients who were treated with *mTOR* inhibitors, whereas a feedback mechanism by *mTORC2* was observed which resulted in *AKT* activation *via* growth factor receptor phosphorylation (*IGF-1R*) (Garay et al., 2015), which provide the foundation to use these inhibitors with *IGF-1R* (Atzori et al., 2011; Chen et al., 2013) or PI3K inhibitors. Thus, precise and timely detection of drug targets or any associated resistance is vital for therapy optimization which can be achieved *via* precision medicine.

## Conclusion

Breast cancer is a very complex and heterogeneous illness with unique molecular and morphological features. Recent advancements in the omics technology have allowed a more precise approach toward the breast cancer classification by understanding the underlying molecular mechanisms. However, there is need for the integration of multiomics approaches which could take omics data from an individual patient and compare it with the databases to guide the strategy for personalized therapy. For management of the breast cancers, the treatment decisions not only depend on the assessment of prognosis factors but also on the evaluation of pathological and clinical factors. An integrated data approach of these multiple factors of breast cancer through multiomics can provide significant insight and hope for making therapeutic decisions. Such personalized therapies will avoid conventional therapeutics where one medicine fits all. It will not only facilitate the patient’s treatment time but also their long-term sufferings as well.
